# Getting up close and personal with UK genomics and beyond

**DOI:** 10.1186/s13073-018-0552-3

**Published:** 2018-05-24

**Authors:** Stephan Beck

**Affiliations:** University College London Cancer Institute, 72 Huntley Street, London, WC1E6BT UK

## Abstract

Stephan Beck discusses recent developments in sharing personal genomes as part of the Personal Genome Project in the UK and globally, and how these efforts are advancing research.

## Introduction

**Fig. 1:**
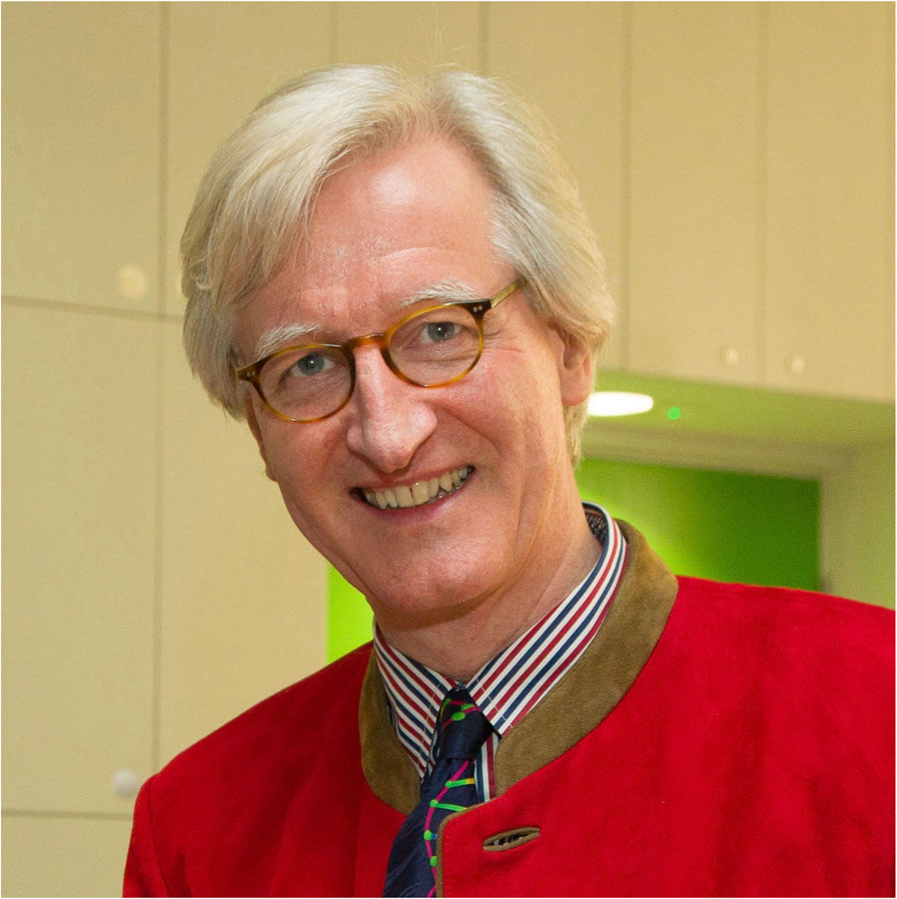
Stephan Beck

Stephan Beck (Fig. 1) is Professor of Medical Genomics at the UCL Cancer Institute and Director of the Personal Genome Project in the UK. He received his PhD in 1985 from the University of Konstanz where he studied DNA structure. After appointments at the Medical Research Council (MRC) Laboratory of Molecular Biology in Cambridge, the Millipore Corporation in Boston and the Imperial Cancer Research Fund in London, he joined the Wellcome Trust Sanger Institute in 1996. During his tenure as Head of Human Sequencing, he played a leading role in the sequencing and analysis of the human and mouse genomes. He has broad interests in the genomics and epigenomics of phenotypic plasticity in health and disease, and in using experimental and computational approaches to advance translational, regenerative and personalized medicine. He is a Fellow of the UK Academy of Medical Sciences and recipient of a Royal Society Wolfson Research Merit Award. In this Q&A, he shares the latest developments in the Personal Genome Project UK (PGP-UK).

## How did you become interested in genomic research and in the sharing of personal genomes?

As a student in 1977, I read the now seminal sequencing papers by the groups of Walter Gilbert and Fred Sanger [1, 2]. Ever since then, I have been tinkering with sequencing technology and using it to study genomes from viruses to humans. Some 20 years later, I was very lucky to become part of the team that sequenced the first human genome and made all the data freely and openly available to everyone, which was a big deal at the time [3]. The legacy of this pioneering effort stimulated biomedical research worldwide and created the foundation for personalized or precision medicine, which I believe will be transformational for our healthcare and life-style decisions in the future.

## What is your role with the personal genome project and what does the collaboration involve?

The Personal Genome Project (PGP) was founded in 2005 by Professor George Church at Harvard University to aid the interpretation and sharing of human genomes (https://pgp.med.harvard.edu/). To facilitate more open data sharing, PGP introduced the concept of open consent and was the first project to provide human genome and trait data under open access. Since then, the project has grown into a global network of currently five PGPs in the US, Canada, the UK, Austria and China under the umbrella of PersonalGenomes.org [4]. All of these PGPs operate independently of each other but jointly advocate for and practise open data access and collaborate on creating publicly shared genome, trait and health data. We believe that this information sharing is critical to scientific progress, but has been hampered by traditional research practices. The PGP approach is to invite willing participants to publicly share their personal data for the greater good.

I am the founding director of the UK branch of the PGP, also known as PGP-UK (https://www.personalgenomes.org.uk/) which started in 2013. Supported by an enthusiastic team, PGP-UK has pioneered the inclusion of methylomes and transcriptomes to complement the analysis of genomes and to gain insights into the epigenetic effects of lifestyle exposures and aging.

## What recent advances have been facilitated or enabled by PGP?

The data and guidelines produced by PGP have already impacted many areas of research and society. Described in over 30 publications to date [4], key areas of innovation include ethics, genomics, method development and citizen science. The most prominent advances have probably been in demonstrating that public sharing of personal omics data is feasible and safe and in establishing a growing network of PGPs that all share the same mission and adhere to the same guidelines [4]:

- Public Data. Participants are invited to share their PGP genomic and trait data publicly in an integrated, publicly accessible format using a Creative Commons (CC0) waiver or equivalent public domain license.
- Non-anonymous. The risks of participant re-identification are addressed up-front, as an integral part of the consent and enrolment process; neither anonymity nor confidentiality of participant identities or their data are promised to research participants.
- Equal access. Participants are provided access to their individual research data in a timely and complete fashion (that is, raw data and not just summary results, where feasible).
- Oversight. Each PGP must at all times maintain current ethics approval or local equivalent, and will work with PersonalGenomes.org [4] to continue to implement identified best practices for responsible public genomics research.
- Not for profit. Each PGP is managed or sponsored by a non-profit organization (or local equivalent). In addition, other than for purposes of reasonable cost recovery, PGPs shall not sell or license participant data or tissues.

## What are the distinguishing features of PGP-UK?

PGP-UK has several distinguishing features that we believe will advance and accelerate personal genomics and medicine. I only highlight the key features here and full details can be found in the PGP-UK pilot study [5].

- First in the EU to use open consent, which allows PGP-UK to link the participants’ multi-omics data with trait, phenotype, environment and health data and to share this information publicly under open access. Open access is one of the key facilitators for developing new technologies and making discoveries.
- First in EU to accept Genome Donations, thereby providing a mechanism for members of the public who had their genomes sequenced elsewhere to share these data publicly. These data may not have been available previously under open access.
- First to report incidental epigenetic findings back to participants along with genetic findings. Like genetic variants, epigenetic variants have been linked to many traits and phenotypes. PGP-UK continues to work closely with the International Human Epigenome Consortium (IHEC) Bioethics workgroup [6] to develop an appropriate framework for reporting such variants.
- First in EU to have facilitated free-of-charge access to cloud-based analysis of personal multi-omics data for non-commercial users on the Seven Bridges Cancer Genomics Cloud [7].
- First to provide a free and open-source app (GenoME) that allows the lay public to explore personal genomes [8]. GenoME introduces the user to genetic and epigenetic variants and showcases examples from volunteer participants in the context of ancestry, traits, phenotypes, aging and environmental exposure using animated graphics and specially composed music based on the variant patterns of the participants [9].

## What projects are the PGP-UK pursuing at the present time?

Building on the achievements of the PGP-UK pilot study described above, we are currently focusing on two exciting new projects. First, the development of a first-generation transcriptome report to complement our genome and methylome reports, which are already being issued to PGP-UK participants. Second, in rolling out the Genome Donation mechanism, we are pioneering a new choice for sharing personal genomes. As sequencing costs continue to fall and more people have their genomes sequenced for one reason or another, we anticipate Genome Donation to become an increasingly popular option and we need to scale our capacity to analyze the data accordingly.

## What impact do you think the PGP-UK will have on our understanding of health and disease?

Like all PGPs, PGP-UK is classified as a research project, so our findings are not suitable or accredited for clinical use. As already demonstrated, PGP data will, however, contribute to advancing both technology and our understanding of genome function in health and disease. On the basis of the positive feedback obtained from PGP-UK participants on our first epigenetic reports, we are encouraged to expand our reporting to include additional exposures as and when it becomes scientifically sound to do so.

## Who can use the PGP-UK’s resources and what are the policies on data availability and sharing?

That’s easy. Once approved by participants, PGP-UK multi-omics data and associated reports will be made available under open access, so they can be used by anyone without restrictions. The open-consent–open-access policies developed by PGP are obviously neither appropriate nor desirable for every project in this space. Nevertheless, many other aspects of responsible data sharing can also be improved and this goal is being explored by IHEC [6], the Global Alliance for Genomics and Health (GA4GH) [10] and others, often in collaboration with PGP.

## How does PGP-UK ensure the accuracy and quality of the data?

Like any other genome project, PGP-UK aims to use best practices to ensure the highest possible accuracy and quality of the data. Nevertheless, in any project of this scale and scope, errors can and will happen. We acknowledge the possibility of errors up-front and openly at every step of the process.

## What are the main challenges PGP-UK faces at the present time?

Obtaining funding is a major challenge. Because PGP-UK is a hybrid between a research and a citizen-science project, it falls into an apparent funding gap between research and community councils and is very much dependent on philanthropic donations. Other challenges are related to the increased complexity and size of the data set. Despite being generated under open access, there is currently no single public database that can accommodate all data types. Increasingly long download times are also becoming problematic. To address these limitations and as already mentioned above, PGP-UK is trialing free (for academia) cloud-based analysis of the PGP-UK pilot study data with a leading provider of a cloud-based computing infrastructure.

## What does the future hold for PGP-UK in the context of other similar initiatives?

For the short-term, PGP-UK certainly has already generated enough data to complete the on-going projects mentioned above. For the longer term and more ambitious analyses, PGP-UK together with the global PGP network will need to find approaches that can accommodate significant project scale-up. This may be possible through the Genome Donation mechanism and/or through the use of blockchain-based technologies that offer optional financial incentives to encourage population-wide participation [11].

## Abbreviations

GA4GH: Global Alliance for Genomics and Health
IHEC: International Human Epigenome Consortium
PGP: Personal Genome Project
